# Volumetric optoacoustic tomography enables non-invasive *in vivo* characterization of impaired heart function in hypoxic conditions

**DOI:** 10.1038/s41598-019-44818-8

**Published:** 2019-06-10

**Authors:** Ivana Ivankovic, Xose Luis Deán-Ben, Hsiao-Chun Amy Lin, Zuwen Zhang, Benjamin Trautz, Andreas Petry, Agnes Görlach, Daniel Razansky

**Affiliations:** 1 Faculty of Medicine and Institute of Pharmacology and Toxicology, University of Zurich, Zurich, Switzerland; 2 Institute for Biomedical Engineering and Department of Information Technology and Electrical Engineering, ETH Zurich, Zurich, Switzerland; 3 Institute for Biological and Medical Imaging, Helmholtz Center Munich, Neuherberg, Germany; 4 Faculty of Medicine, Technical University of Munich, Munich, Germany; 5 Experimental and Molecular Pediatric Cardiology, German Heart Center Munich at the Technical University of Munich, Munich, Germany; 6 DZHK (German Centre for Cardiovascular Research), Partner site Munich, Munich Heart Alliance, Munich, Germany

**Keywords:** Photoacoustics, 3-D reconstruction

## Abstract

Exposure to chronic hypoxia results in pulmonary hypertension characterized by increased vascular resistance and pulmonary vascular remodeling, changes in functional parameters of the pulmonary vasculature, and right ventricular hypertrophy, which can eventually lead to right heart failure. The underlying mechanisms of hypoxia-induced pulmonary hypertension have still not been fully elucidated while no curative treatment is currently available. Commonly employed pre-clinical analytic methods are largely limited to invasive studies interfering with cardiac tissue or otherwise *ex vivo* functional studies and histopathology. In this work, we suggest volumetric optoacoustic tomography (VOT) for non-invasive assessment of heart function in response to chronic hypoxia. Mice exposed for 3 consecutive weeks to normoxia or chronic hypoxia were imaged *in vivo* with heart perfusion tracked by VOT using indocyanide green contrast agent at high temporal (100 Hz) and spatial (200 µm) resolutions in 3D. Unequivocal difference in the pulmonary transit time was revealed between the hypoxic and normoxic conditions concomitant with the presence of pulmonary vascular remodeling within hypoxic models. Furthermore, a beat-to-beat analysis of the volumetric image data enabled identifying and characterizing arrhythmic events in mice exposed to chronic hypoxia. The newly introduced non-invasive methodology for analysis of impaired pulmonary vasculature and heart function under chronic hypoxic exposure provides important inputs into development of early diagnosis and treatment strategies in pulmonary hypertension.

## Introduction

Pulmonary hypertension (PH) is a disorder characterized by pulmonary vascular remodeling, right ventricular hypertrophy and increased pulmonary arterial pressure. PH has been associated with various disorders while, according to the recent WHO classification, it is regarded as a separate entity when associated with hypoxia or chronic diseases of the respiratory system^1^. The latter include chronic obstructive pulmonary disease (COPD), interstitial lung diseases, sleep disordered breathing, but also chronic exposure to high altitude and some rare neonatal diseases^2,3^.

Murine models have been widely used to gain deeper insight into lung-heart interactions under chronic hypoxic conditions^4^. The hypoxia-inducible factor (HIF) has been highly implicated in the development of PH^5^ where several preclinical studies focus on the molecular mechanisms of HIF and its role in PH^6–9^. However, there is a lack of direct functional studies of the heart in response to chronic hypoxia. Methods for assessing pulmonary and cardiac structural alterations in PH are usually limited to histopathological *ex vivo* analyses looking at right ventricular (RV) hypertrophy and pulmonary vasculature changes. RV catheterization is an invasive *in vivo* procedure to measure pressure differences as a surrogate parameter of increased pulmonary arterial pressure^10–13^. Yet, the majority of the analytical methods interfere with integrity of the heart, which calls for introduction of new methods for direct *in vivo* assessment of cardiopulmonary coupling in small animal models.

*In vivo* imaging of the murine heart is challenging due to its small size and rapid motion^14^, which imposes hard requirements on the spatial and temporal resolution of non-invasive imaging modalities to accurately capture a heart volume of less than a cubic centimeter beating at a 400–600 cardiac cycles per minute. Even though cardio-respiratory gating in magnetic resonance imaging (MRI) and X-ray computed tomography (CT) may enable characterizing some of the *in vivo* functional cardiac parameters, those methods are generally ill-suited for cardiac imaging due to insufficient temporal resolution when performing true 3D whole-heart imaging at high spatial resolution^15,16^. To this end, ultrasound (US), and more recently ultrafast US, are arguably the most suitable modalities for cardiac imaging in murine models. US enables discerning anatomy and measuring important physiological parameters, such as blood flow^17^. Yet, it is generally not suitable for measuring some of the key functional parameters in the entire heart volume in 3D, in particular for analysis of right ventricular size within murine models. To our knowledge, very few methods generally exist for non- or minimally-invasive imaging and assessment of the effects of exposure to chronic hypoxia in pre-clinical models.

Optoacoustic (OA) imaging is becoming an increasingly powerful tool in pre-clinical research, in particular for *in vivo* cardiac imaging in murine models, for a number of reasons: (1) the high number of effective voxels rendered by state-of-the-art systems allows for imaging the whole murine heart with high spatial resolution using stationary matrix detection arrays; (2) optical contrast allows for blood perfusion monitoring^18^, and (3) the high temporal resolution in 3D facilitates analyzing functional parameters of the living murine heart on a beat to beat basis^19^. An important functional parameter that can be measured with OA via injection of a contrast agent is the pulmonary transit time (PTT). The latter has been shown to significantly decrease in infarct murine models and hence can serve as an important indicator of heart function^19^. Another key feature of volumetric optoacoustic tomography (VOT) is the ability of imaging the entire heart with a single laser pulse. This is crucial to characterize potential delays in cardiac activation across different regions, e.g. during arrhythmic events. In this work, we demonstrate the capabilities of a recently developed real-time three-dimensional OA imaging system for analyzing heart function *in vivo* and non-invasively in chronic hypoxic murine models.

## Results

### Volumetric optoacoustic tomography of the murine heart

**Figure 1:**
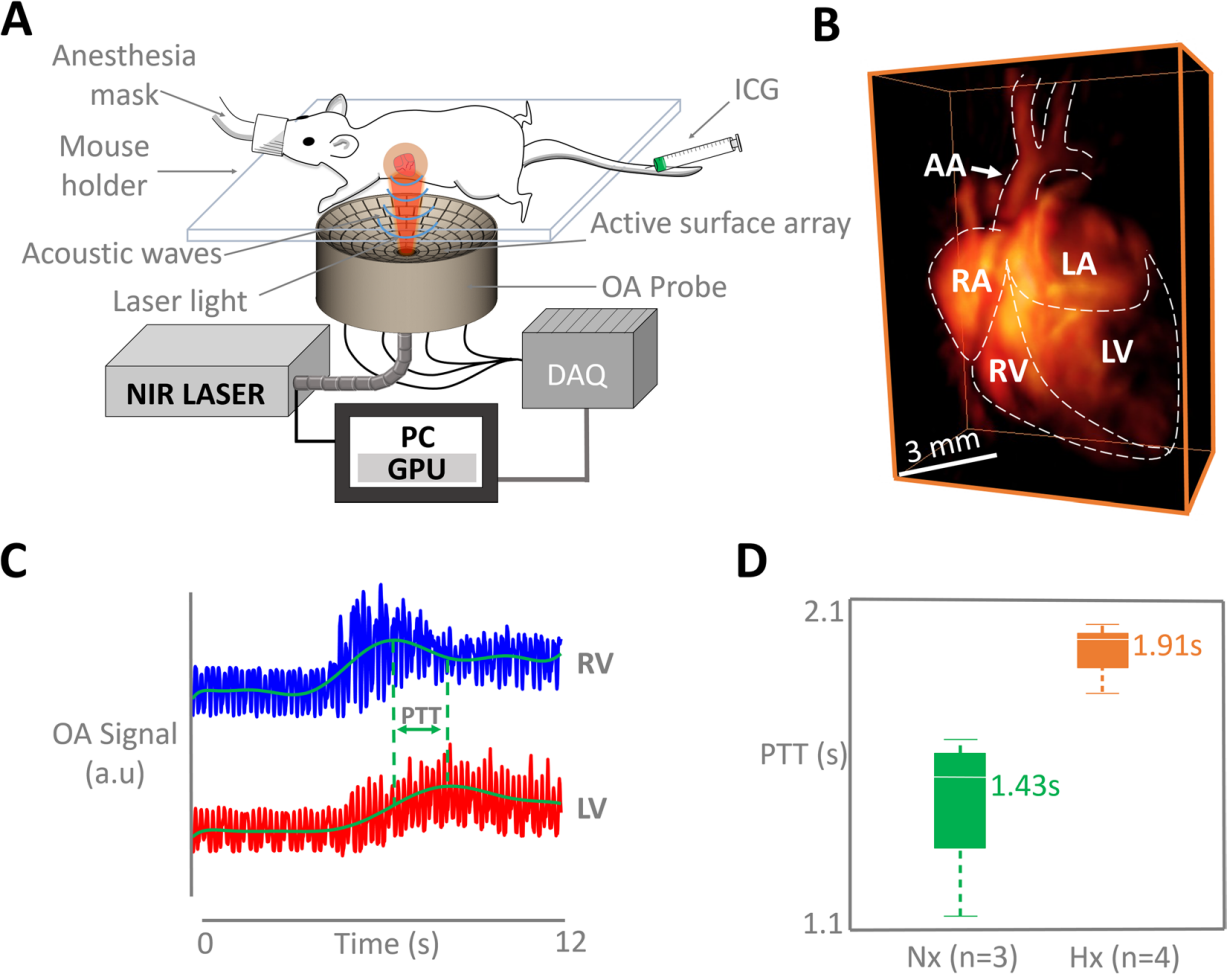
The experimental protocol. (**A**) Non-invasive imaging procedure of the murine heart with volumetric optoacoustic tomography. (OA; optoacoustic, ICG; indocyanine green, NIR; near-infrared, PC; personal computer, GPU; graphics processing unit, DAQ; data acquisition system). (**B**) 3D view of the optoacoustic image of the murine heart reconstructed with a single laser pulse (AA - aortic arch, RA - right atrium, LA - left atrium, RV - right ventricle, LV - left ventricle). (**C**) Temporal profiles of the optoacoustic signal intensities in two voxels in RV and LV, as indicated in (B). (**D**) The pulmonary transit time (PTT) is calculated as a difference in time of arrival of the contrast agent, i.e. time difference corresponding to the maximum signal values in the RV and LV (Hx; mean-1.91 [1.7386–2.02] s versus Nx; mean-1.43 [1.0602–1.64] s, *P* < *0*.0023).

The VOT imaging setup was optimally designed for *in vivo* murine heart imaging (Fig. 1A). The dedicated design consists of a spherical array transducer which was held pointing upwards for optimal OA signal detection of the heart, a fiber bundle for light illumination, a fast-tuning optical parameter oscillator laser and a data acquisition system for simultaneous OA signal detection for all elements of the array. Mice which have been exposed to chronic hypoxia for 3 weeks (n = 4) and normoxic counterparts (n = 3) were anesthetized and laid on the transducer with the chest facing down (See Methods section for a detailed system description). High-frame-rate images of the beating murine heart were acquired with the VOT system, specifically designed for high performance pre-clinical cardiac imaging (Fig. 1B). The VOT data was acquired for each ICG injection for a total duration of 50 s (5000 frames), which is a sufficient amount of time to track blood flow through the pulmonary circuit.

### Pulmonary transit time

The PTT was measured as the difference between the time points corresponding to the maximum signal peaks for the right (RV) and left ventricles (LV), corresponding to the time of appearance of the ICG bolus (Fig. 1C). Boxplots of the measured PTT values for hypoxia-treated (n = 4, 6 injections altogether) and normoxic models (n = 3, 6 injections altogether) are presented in Fig. 1D. A t-test was carried out to analyze the difference between the observed PTT values. The PTT values measured from the hypoxic models were significantly longer than those obtained for the normoxic models (mean-1.91 [1.7386–2.02] s versus mean-1.43 [1.0602–1.64] s, *P* < 0.0023). This clear difference in PTT between hypoxia-treated and normoxic mice strongly suggests that chronic hypoxia affects cardiac function and/or pulmonary hemodynamics.

### Immunohistochemistry and right ventricular hypertrophy

**Figure 2:**
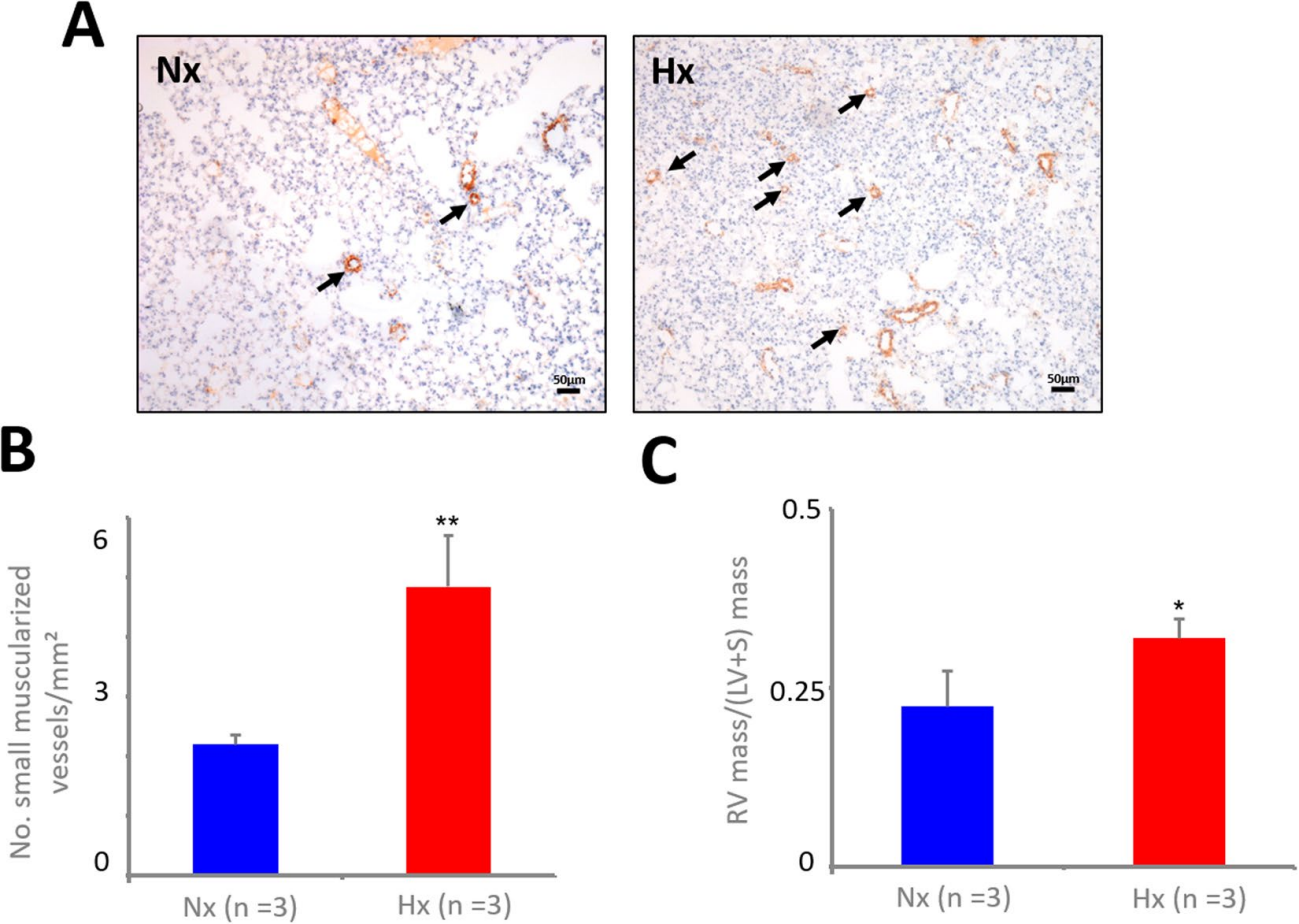
Staining for α-smooth-muscle actin of murine lungs. Formalin fixed and paraffin embedded (FFPE) lung sections derived from normoxic (Nx) or hypoxic (Hx) mice were stained for α-smooth-muscle actin and the number of muscularized small vessels (<30 µm) was counted. (**A**) Small muscularized vessels are indicated with arrows. (**B**) Graph shows the number of small muscularized vessels per mm^2^ lung tissue, assembled from three regions of interest per lung (n = 3, **p < 0.01 Hx vs. Nx). (**C**) The Fulton index as measure of right ventricular hypertrophy was determined as ratio between mass of the right (RV) and left ventricle (LV) with septum (S) (n = 3, *p < 0.05 Hx vs. Nx).

Characteristically, exposure to chronic hypoxia results in a vasoconstrictor response as well as in muscularization of small pulmonary vessels indicative of pulmonary vascular remodeling leading to an increase in pulmonary vascular resistance and right ventricular hypertrophy. As evinced by staining for α-smooth muscle actin of lung sections (Fig. 2A), the number of small muscularized pulmonary vessels was significantly increased in lung sections from hypoxic mice in comparison to normoxic mice (Fig. 2B), indicating pulmonary vascular remodeling. Subsequently, the masses of the right and left ventricle including septum were measured and the Fulton index was determined as a measure of right ventricular hypertrophy. Compared to the normoxic mice, the Fulton index was elevated in hypoxic mice indicating right ventricular hypertrophy (Fig. 2C).

### Heart beat characterization

**Figure 3:**
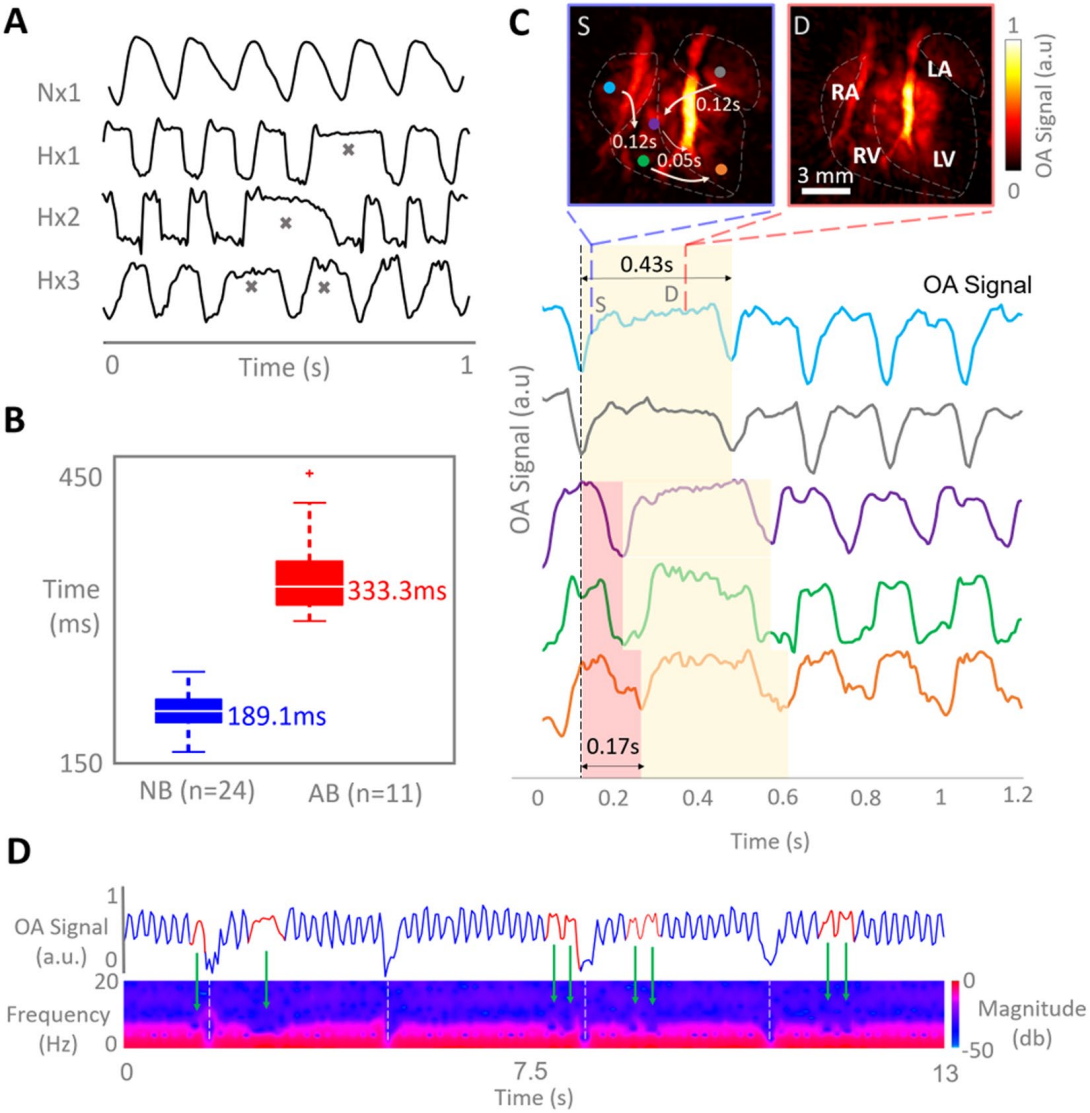
Optoacoustic characterization of impaired heart function in hypoxic models. (**A**) Examples of time-lapse optoacoustic signal intensity profiles for selected voxels in the heart of normoxic and hypoxic mice (n = 4). Irregular heart beating events are marked with grey crosses in the time traces for hypoxic models. (**B**) Boxplots of the measured cycle period for normal versus abnormal beating cycles (NB - normal beating, AB - abnormal beating). (**C**) Volumetric mapping of the heart mechanical motion and onsets of the irregular beats, where the colored circles in the heart in systolic phase (S – blue box) correspond to the colored OA signal profiles below. The red shade in the profiles indicate heartbeat onset at varying locations and the yellow shade indicates the duration of the heartbeat. The diastolic phase of the heart (D-red panel) is the heart phase present for the majority of the heartbeat (RA – right atrium, LA, left atrium, RV – right ventricle, LV – left ventricle, S-systole, D-diastole). (**D**) Short-time Fourier transform (STFT) of the temporal OA signal profile in a selected voxel in hypoxic heart, where blue indicates normal beating periods and red indicates irregular beating periods. Green arrows identify the areas of abnormal beat periods in the time series and the areas of low frequency acquired from STFT. White dashed lines indicate breathing events.

Due to the excellent temporal resolution of the VOT system, it was possible to characterize the heart motion on a beat-by-beat basis. Heart rate variability was clearly identified within the hypoxic models (n = 3 (3 of 4)) in VOT image sequences of 500 frames (100 frames per second), whereas only periodic cycles were recorded in normoxic models (Fig. 3A). The irregular heartbeats are also clearly visible in the supplementary video of the reconstructed VOT image sequences available in the online version of the journal. A t-test analysis revealed that the irregular cycles, marked with grey crosses in Fig. 3A, have a length of 333 [282–437.3] ms versus 189 [145–229] ms, *P* < 0.001 (Fig. 3B). The real-time 3D imaging capability of VOT allows for mapping the mechanical motion globally throughout the heart by plotting time-lapse OA signal intensity profiles in different locations within the heart wall (Fig. 3C). The profiles indicate that the length of the irregular heart cycle is approximately twice the duration of the regular cycles. This may be attributed to a steady sinus rate and impaired atrioventricular conduction or ‘heart block’ rather than a supra-ventricular arrhythmia^20^. Atrial deformations may additionally be produced during ventricular pauses, which may become visible if the resolution of the VOT system is enhanced by using a higher frequency array. The cycle length lasts for approximately 430 ms, by which ventricular activity is primarily in diastole (D). The irregular cycles are also clearly visible in the supplementary videos of the reconstructed VOT image sequences available in the on-line version of the journal. Figure 3D shows the short-time Fourier transform (STFT) analysis carried out on image data from a hypoxia-treated murine model revealing multiple irregularities throughout cycle length. By calculating the frequency spectra of the time profiles as a function of time, the STFT facilitates detecting changes of heart rate over time, corresponding to arrhythmic events as areas of lower frequencies of the time-series (Fig. 3D). The green arrows in Fig. 3D represent irregular cycles, while the white dashed lines indicate breathing periods. The profile in Fig. 3D show that the peaks of normal contractions before and after arrhythmic events follow a defined periodicity, which further suggests failure in atrio-ventricular conductivity.

## Discussion

Pre-clinical animal models are commonly used to investigate the pathophysiology of pulmonary hypertension and potential therapeutic interventions. Exposure to chronic hypoxia over 3 weeks characteristically leads to pulmonary hypertension in mice. To this end, pre-clinical assessment of PH has been largely limited to *ex vivo* or invasive procedures that interfere with the integrity of the heart tissue, hampering an accurate assessment of heart function within an intact living organism. Development of new imaging approaches is thus crucial for comprehensive understanding of the scope of PH in an *in vivo* environment.

In this study, we have examined the potential of VOT for assessing *in vivo* heart function in murine models of chronic-hypoxia-induced PH. The high temporal resolution of the imaging system enables tracking fast perfusion of contrast agents and estimation of the pulmonary transit time (PTT), a valuable capacity for functional assessment of the heart and pulmonary circuit dynamics. PTT has previously been shown to serve as an accurate indicator of heart performance, specifically left ventricular performance under pathophysiological conditions in the murine heart^19^. In this study, we have shown that the PTT in murine models exposed to chronic hypoxia was considerably longer than in normoxic models. As the PTT is determined by cardiac function and pulmonary hemodynamics, these findings point to deteriorated cardio-pulmonary function in response to chronic hypoxic conditions. In fact, as indicated in this study, chronic hypoxia results in pulmonary vascular remodeling and right ventricular hypertrophy. This has not only been associated with an increase in pulmonary vascular resistance and right ventricular pressure due to increased afterload in chronic hypoxic mice^21,22^, but has also been associated with a decreased ability of conductance vessels to store and deliver the entire stroke volume of the right ventricle and ultimately resulting in a loss of pulmonary flow during diastole^23^. PTT values (or CPTT - Cardiopulmonary Transit Times) have been previously studied in humans by using the first pass radionuclide cardiography technique^24^. In support of our studies on hypoxia-induced PH in mice, longer PTT values were shown in patients with pulmonary hypertension^25,26^. PTT is becoming increasingly recognized as an important marker for cardiopulmonary function, where very recent studies have been evaluating the PTT in patients using MRI and US^27^. At present, pulmonary hypertension in mice is mostly characterized by invasive measurement of right ventricular pressure, and *ex vivo* histopathological analysis of pulmonary and cardiac tissues. Very recently, MRI and US has been applied to evaluate pulmonary hypertension in the more severe hypoxia/Sugen5416 murine model by measuring right ventricular ejection fraction^28^ However, in mice PTT was only determined invasively using microangiography^29^. VOT enables measuring the PTT *in vivo* in mice, thus offering a new efficient and non-invasive tool to monitor heart remodeling and pulmonary circuit dynamics in models of pulmonary hypertension.

Arrhythmic events, in particular atrial fibrillation or flutter have been frequently observed in patients with pulmonary hypertension or COPD^30^. However, heart rate or arrhythmia have not been well documented in the adult chronic hypoxia murine model. Here we show that irregular heart cycles were present in mice exposed to chronic hypoxia as detected in the time profiles of the VOT data that represented mechanical motion of the heart in three dimensions. Although actual blood pressure values cannot be extracted from the VOT data, the non-invasively recorded OA signal intensity changes extracted at given spatial locations, plotted similar patterns to pressure waveforms usually extracted via catheterization^31^. This is expected considering that pressure changes in the heart chambers induce a displacement or strain in the heart walls, which are easily detectable in the OA signal intensity profiles. In catheterization procedures, pressure waveforms can only be measured at a specific location of the heart, which generally hinders measuring the delays between mechanical activation across different heart regions. With the suggested VOT approach, the OA signal intensity changes can be simultaneously obtained from multiple locations in the heart, which enables readily identifying such delays and could serve as an alternative method to strain imaging which also maps mechanical activation across the heart. The preliminary results presented here on cycle variability have promoted future studies on this topic using VOT in order to fully understand the effect of chronic hypoxia on the electromechanical activity of the heart, where ECG and VOT data would be directly compared.

In conclusion, *in vivo* simultaneous detection of two impaired heart functions have been demonstrated with VOT in the murine model of chronic-hypoxia-induced pulmonary hypertension. The PTT and heart rate were both altered in hypoxic hearts compared to normoxic hearts. Overall, VOT has been suggested as a method offering new capabilities for *in vivo* volumetric beat-by-beat characterization of cardio-pulmonary function in murine models of pulmonary hypertension, not attainable with existing approaches.

## Methods

### Animal models

All animal procedures were approved by the local legislation on protection of animals (Government of Upper Bavaria, Munich, Germany under animal protocol reference number 55.2-1-2532-50-12) and conducted in accordance with the European directive 86/609/EEC and internal regulations of the Technical University of Munich and Helmholtz Centre Munich. Mice (129S/Sv/C57BL6 mixed background) were maintained for 21 days either under normoxic conditions (n = 3) or under hypoxic conditions (10% oxygen, n = 4) in a custom-built normobaric chamber, as described previously^21^.

### Animal handling

All mice were anesthetized with approximately 2% isoflurane – oxygen medical mix (~0.81 L/min gas flow) for *in vivo* imaging of the heart. The fur covering the region of interest was initially clipped and then completely removed with hair removal cream. During imaging, the anesthetized mice were placed on top of a solid agar matrix filling the volume enclosed by the spherical array (Fig. 1A). Warmed ultrasound gel was further used between the tissue surface and the agar matrix for optimum acoustic coupling and maintaining homeostasis. Approximately 15 sec after beginning of the image acquisition each mouse was intravenously injected with 100 nmol/L of indocyanine green (ICG) contrast agent (Profiplus Bvba, Kortessem, Belgium) diluted in 50 µl saline solution. Mice were then injected a second time 10 minutes after the first injection.

### Volumetric optoacoustic tomography (VOT) of the murine heart

High-frame-rate images of the beating murine heart were acquired with a VOT system specifically designed for high performance pre-clinical cardiac imaging (Fig. 1B)^18,32^. The imaging system consists of a spherical array transducer (Imasonic Sas, Voray, France) composed of 512 piezoelectric elements with 5 MHz central frequency and >80% detection bandwidth. The array provides 140° solid angular coverage with 40 mm radius. The large angular coverage of the array reduces limited-view effects and further enables an improved sensitivity and deeper penetration. In the experiments, the spherical array was held pointing upwards (Fig. 1A). The spherical volume enclosed by the spherical aperture was filled with clear agar (3 w/v% concentration), which provided acoustic coupling while being transparent for light. Short light pulses (<10 ns) at 800 nm wavelength and 100 Hz repetition frequency were generated by a fast-tuning optical parameter oscillator laser (Innolas Laser GmbH, Krailling, Germany) and guided via a fibre bundle (Ceram Optec GmbH, Bonn, Germany) through the center of the transducer array. The 800 nm wavelength corresponds to the absorbance peak of ICG. The OA signals for all elements of the array were simultaneously sampled by a custom-made parallel data acquisition system (Falkenstein Microsysteme GmbH, Taufkirchen, Germany). Volumetric images were reconstructed on the fly for each laser pulse by a graphics processing unit-based 3D back-projection reconstruction algorithm, which enabled real-time preview during the experiments and facilitated correct positioning of the animal^33^. After correct positioning, VOT data was acquired for a total of 5000 frames and 3D images were later reconstructed offline in a volume of 12 × 12 × 12 mm^3^ (120 × 120 × 120 voxels). All processing steps were performed in MATLAB (MathWorks Inc, Natick, USA).

### Pulmonary transit time (PTT)

The method for extracting the PTT values from the heart image sequence has been previously described^18^. In short, the PTT was measured as the difference between the time points corresponding to the maximum signal peaks for the right (RV) and left ventricles (LV), corresponding to the time of appearance of the ICG bolus (Fig. 1C). The PTT was measured for both hypoxic and normoxic models, followed by statistical analyses. Voxels in the RV and LV were identified in the VOT images using a 4-dimensional viewing toolbox in Matlab.

### Cardiac cycle characterization

OA signal intensity profiles were extracted from different locations in the LV and RV, the left atria and right atria as well as the aortic arch by selection of voxels in the images. These cycles were used for characterizing the heart rate as well as irregularities corresponding to arrhythmias. The latter were identified when the length of a single cycle significantly exceeded the average cycle length of a normal periodic rhythm. Also, the spectrogram of the cardiac cycle was calculated as the short-time Fourier transform (STFT) of the signals. The STFT was used to resolve the frequency content at specific time points and track it over time. Abnormal beating events are expected to result in a lower frequency distribution in the STFT as opposed to the normal beating rhythm.

### Immunohistochemistry and Fulton index

Hypoxia-induced pulmonary vascular remodeling was validated by immunohistochemistry, as described previously^21^. Briefly, lung tissue samples were immersed in 10% buffered formalin solution for 48 h and subsequently embedded in paraffin (FFPE). FFPE lung sections were stained with an antibody against α-smooth muscle actin (clone 1A4; DAKO, Hamburg, Germany). The slides were heated at 60 °C for 1 h before rehydration in a series of alcohol solutions of decreasing alcohol concentration. The endogenous peroxidase activity was quenched in 1% hydrogen peroxide solution in methanol. The hydration process was completed by rinsing in DAKO wash buffer. Sections were heated in a water bath at 90 °C while submerged in antigen retrieval pH 9 epitope retrieval solution (DAKO) for 30 min. They were subsequently blocked in blocking reagent for 1 h, and then incubated with the antibody 1:100 diluted in M*O*M diluent (Vector M*O*M kit) (Vector Laboratories, Burlingame, CA) for 1 h at room temperature in a humidity chamber. The sections were washed in DAKO wash buffer and the secondary antibody was applied (anti-mouse IgG in dilution 1:250). The avidin–biotin complex (Vectastatin Elite Kit, Vector Laboratories) was applied to the slides for 30 min at room temperature. The chromogenic reaction was performed with diaminobenzidine (DAB; DAKO) for 5 min at room temperature. Slides were counterstained with Mayer’s hematoxylin for 30 s (Merck, Darmstadt, Germany), dehydrated in an ascending alcohol concentration, and mounted with Entelan (Merck). Positive and negative controls were included with each run. For evaluation of each lung, small α-smooth muscle actin positive vessels less than 30 µm were identified in three randomly selected regions of interest covering an area of 1 mm^2^ each^22^.

To determine the Fulton index as a measure of right ventricular hypertrophy, the right ventricle was separated from the left ventricle and septum, and masses were determined.

## Supplementary information

Video 1
